# Introduction of a Simple Technique for Partial Splenectomy in Multiple Trauma Patients

**DOI:** 10.5812/ircmj.9072

**Published:** 2013-12-05

**Authors:** Mehdi Eskandarlou, Amir Derakhshanfar

**Affiliations:** 1 Department of Surgery, Hamedan University of Medical Sciences, Hamedan, IR Iran

**Keywords:** Wounds and Injuries, Spleen, Splenectomy

## Abstract

**Background:**

The spleen is the most commonly injured intraperitoneal organ in multiple trauma patients. Total splenectomy results in immunodeficiency and predisposes patients to certain infections.

**Objectives:**

Performing partial splenectomy with a safe, simple, and definite technique in trauma patients with hemodynamic instability and accompanying intra-abdominal injury could play an important role in the preservation of immune function and reducing morbidity.

**Patients and Methods:**

From 2006 to 2009, a total of 20 patients underwent partial splenectomy, at Mobasher and Be’sat hospitals. Patients with splenic injuries of up to stage IV and grade 3 shocks underwent partial splenectomy. The operations were performed without vascular isolation and by wedge resection of the injured splenic tissue and repair with chromic 2/0 sutures in two rows. Three months later, patients were evaluated by a Tc99 liver-spleen scan, complete blood count, and blood smear.

**Results:**

There were 16 male and four female patients with an age range of 4 to 54 years old. Ten patients had additional intra and extra abdominal injuries. The salvaged spleen tissue was approximately 30% in nine patients, 40 to 50% in two, and more than 50% in another nine patients. The operation time was less than three hours and hospital stay was 3 to 15 days for 90% of the patients. No complications occurred after the surgery or during the follow up. For all patients, the complete blood count, peripheral smear, and liver-spleen scan were normal after six months.

**Conclusions:**

Partial splenectomy with preserving at least 30% of the splenic tissue can be performed for trauma patients using wedge resection of the injured splenic tissue and repair by chromic 2/0 sutures in two rows. Using this technique, there is no need for vascular isolation or hemostatic materials. Splenic function is presented and associated intra and extra abdominal injuries are not contraindications for partial splenectomy.

## 1. Background

The spleen is the most commonly injured intra-abdominal organ, during both blunt and penetrating traumas and accounts for up to 45% of all visceral injuries (1, 2). The spleen plays an important role as a part of the reticuloendothelial system (2, 3). It is also a major part of the immune system, after initially serving as a hematopoietic organ during early gestation (4). The spleen collects damaged red blood cells, particulate matter, and bacteria from the bloodstream. It also serves as a reservoir for up to 25 % of the circulating platelets (4).

Injuries of the spleen can be managed by one of three methods including splenectomy, splenorrhaphy, or non-operative observation (5). For decades, splenectomy was considered the safest approach for injured spleen (6). However, patients that undergo splenectomy become subject to an acquired immunodeficiency state and are therefore at increased lifetime risk for certain infections (7-9). Serious infections, known as overwhelming postsplenectomy infections occur in 1.4% of all such cases (7-9). The relative risk of fatal infection in splenectomized patients is 200 times higher than the general population (10). Such infections can result in death in less than 6 hours.

Because of the serious complications of splenectomy, nowadays splenic preservation has been considered as the preferred treatment for the injured spleen (11-13). In addition, animal experiments have demonstrated that partially splenectomized cases are normally protected against sepsis (14).

## 2. Objectives

Considering the important role of the spleen and also recent improvements in surgical techniques, it is recommended to perform partial rather than total splenectomy especially in children, following spleen injuries to prevent consequent immunosuppression. To evaluate the efficacy of partial splenectomy and also introduce a new surgical technique for patients with blunt or penetrating spleen trauma, this study was conducted in Hamedan, a major city in the west of Iran.

## 3. Patients and Methods

### 3.1. Patients

Twenty patients with multiple traumas including injury to the spleen were included in this interventional study from Shahid Mobasher and Be’sat hospitals located in Hamedan, west of Iran from 2006 to 2009. Ethical approval was received with code P/16/35/228 on the 23rd of August 2011. Spleen injury was diagnosed by physical examination and paraclinical findings preoperatively or during exploratory laparatomy.

A complete blood count was obtained from all patients and 150 mg/kg of cephalothin was prescribed as a prophylactic antibiotic prior to surgery. Partial splenectomy was performed for patients who had a stable hemodynamic situation and there were no signs of severe shock (stage IV). In addition, the injury was graded intraoperatively by examining the spleen itself and its hilar area, and partial splenectomy was performed only for patients with grade IV spleen injury or lower (Table 1) (4).

The purpose of the operation was to salvage at least 30% of the viable spleen tissue. Regarding ethical consideration, all patients with diagnosis of splenic injury either preoperatively or during exploratory laparatomy for other reasons were evaluated for life threatening risk factors that preclude using our technique. Therefore the patients who had multiple organ injuries with high risk of bleeding, massive contamination and intraperitoneal infection or brain damage or a Glasgow coma scale lower than 12 were excluded from the study. The patients were guaranteed and we covered all extra charges related to complications of this surgical technique such as the need for preoperation to control bleeding. Also the patients and their families were informed about the surgical procedures and subsequent follow ups, and a written consent was obtained prior to the surgery.

**Table 1.** Spleen Injury Stages

Stage	
**Grade I**	Subcapsular hematoma <10% surface area, capsular tear < 1 cm in depth.
**Grade II**	Subcapsular hematoma, nonexpanding, 10 to 50% surface area; intraparenchymal hematoma, nonexpanding, <2 cm in size; capsular tear, active bleeding, 1 to 3 cm size, does not involve trabecular vessels.
**Grade III**	Subcapsular hematoma, >50% surface area or expanding; intraparenchymal hematoma, >2 cm in size or expanding; laceration >3 cm in depth or involving trabecular vessels.
**Grade IV:**	Ruptured intraparenchymal hematoma with active bleeding; laceration involving segmental or hilar vessels producing major devascularization (>25% of spleen).
**Grade V**	Shattered spleen; hilar vascular injury that devascularizes the spleen.

### 3.2. Methods

**Figure 1.**
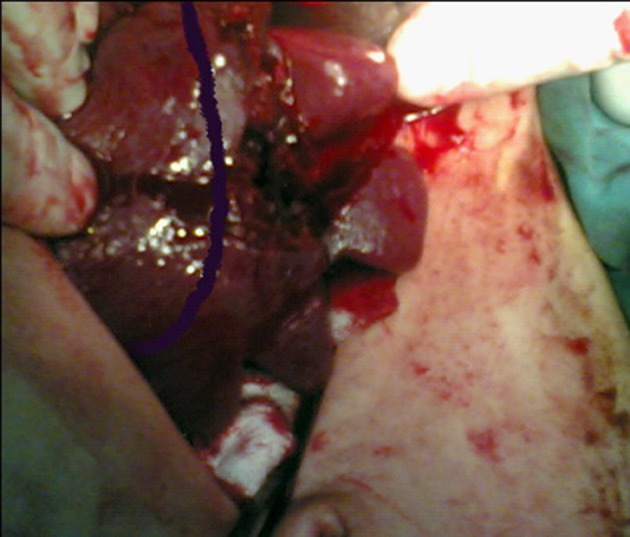
Stage IV Splenic injury in a 34 Year Old Patient

After systematically evaluating the entire abdominal cavity, ligamentous and peritoneal adhesions were separated and the spleen was carefully mobilized. Bleeding was controlled manually by compressing hilar vessels. The purpose of the operation was to salvage at least 30% of the viable splenic tissue in the upper and lower margins and to resect the ischemic and damaged spleen tissue. Viable splenic tissue was marked with a 1 cm margin, proximal to the injured area, by methylene blue (Figure 1). 

**Figure 2.**
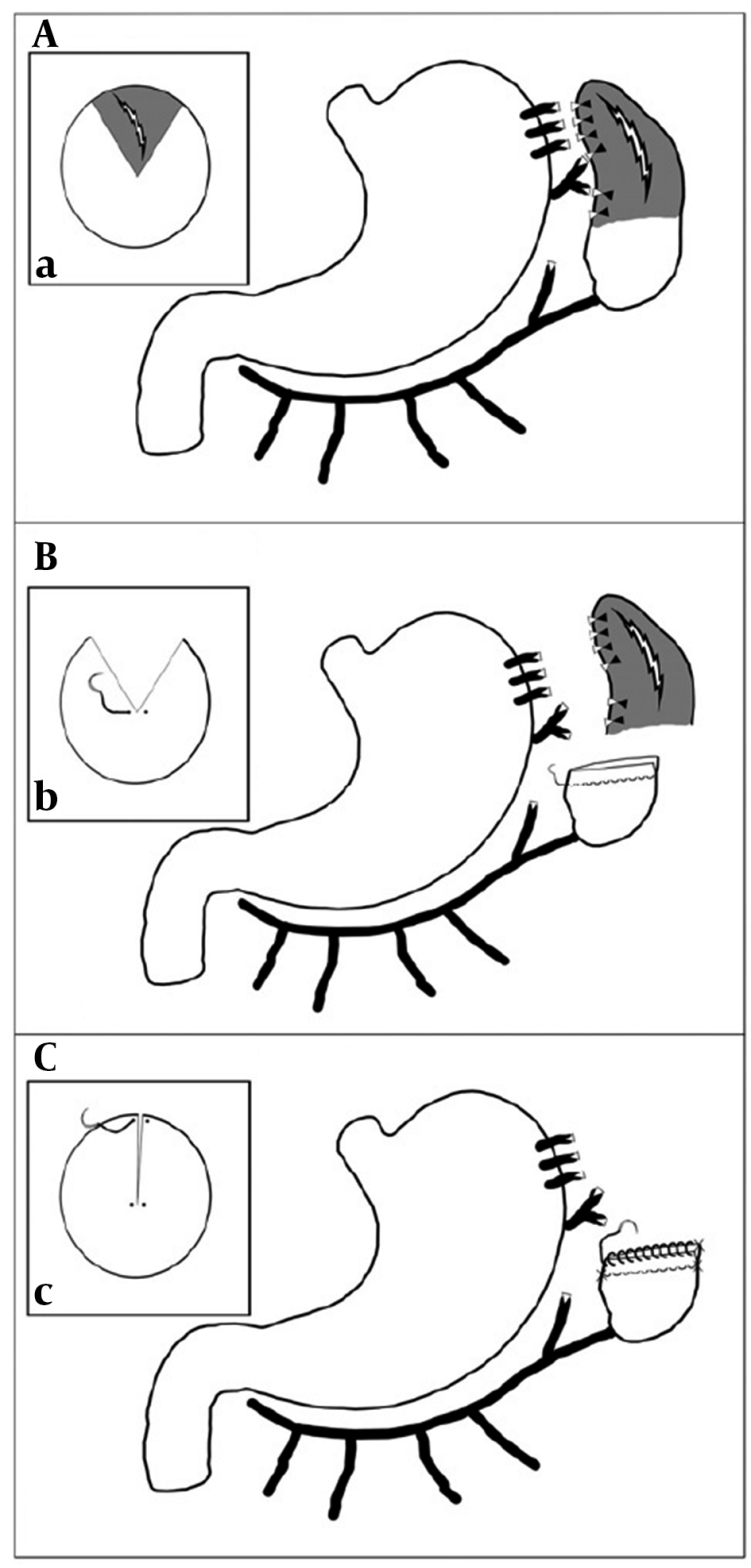
Schematic Representation of Partial Splenectomy (A) Splenic injury to the upper pole with central portion ischemia; (B) Partial splenectomy using wedge or V-shaped resection of the ischemic areas, applying the first layer of hemostatic sutures, and repair of the remaining lower pole on the base of the left gastroepiploic artery with continuous horizontal mattress sutures; (C) Applying the second layer repair of the remaining lower pole with chromic 2/0 simple continuous sutures; (a, b, and c) The lateral view of the injured spleen

Using a blunt needle and 2/0 chromic catgut, a row of continuous mattress sutures was applied along the marked line on the spleen (Figure 2). 

**Figure 3.**
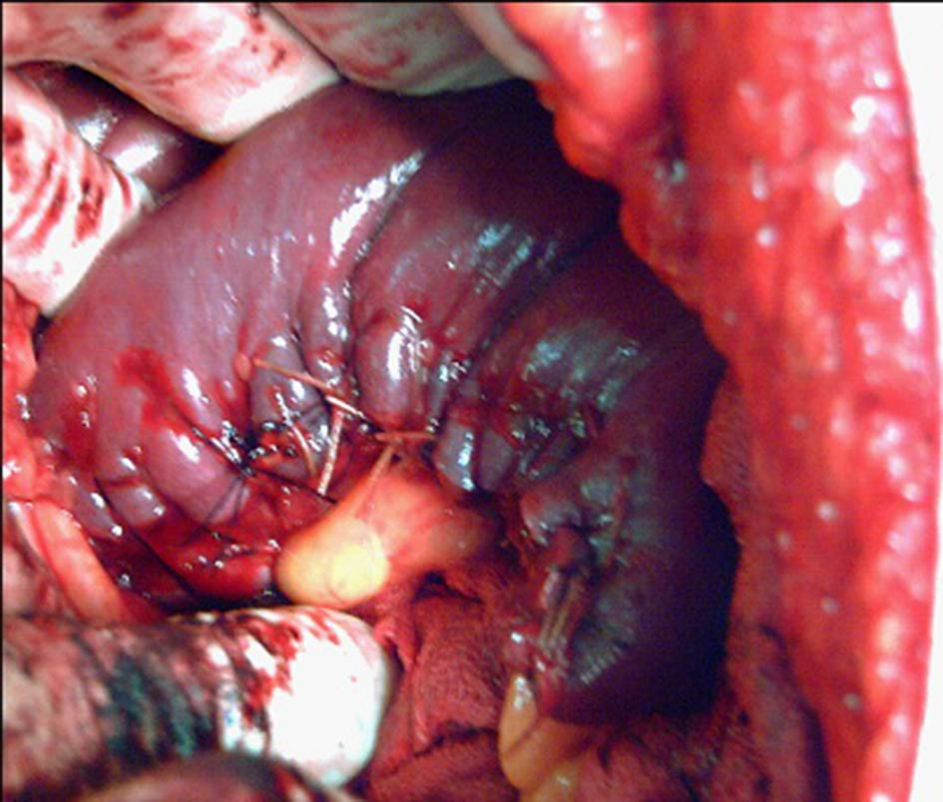
Partial Splenectomy in a 34 Year Old Patient

Joining the two ends of the mattress sutures, the damaged spleen tissue was resected with a 1 cm margin distal to the marked area in a wedge shaped (V-shaped) pattern. Then, the second row of sutures using 2/0 chromic catgut and a simple continuous technique was applied 3 mm distal to the first row and close to the resected area, joining the two resected edges to form a new margin similar to the normal spleen margin (Figure 3). In cases of large and thick spleens (especially in adults) a 5 cm blunt liver needle was used. 

After applying the second row of sutures, the assistant surgeon gradually released the spleen and checked the sutured edges to identify possible bleeding areas. Hemostasis would be established using interrupted mattress sutures with 2/0 chromic catgut if bleeding was observed. The same procedure was performed when a large area of the upper or lower poles and central areas had been injured. When the upper pole of the spleen was salvaged, the short gastric artery and a long branch of the splenic artery were saved and in cases of lower pole salvaging, the left gastro epiploic artery and a long branch of the splenic artery were kept. Other hilar vessels that supplied the injured areas were ligated using sutures after resecting the damaged tissue. Finally, a Penrose drain was inserted under the spleen in the left upper quadrant.

In this surgical procedure no vascular isolation technique such as cautery, ligature, stapling, laser, or omentum pad was used to control bleeding. The Penrose drain was removed three days after the surgery in all patients. All patients whose splenic injury was diagnosed intraoperatively received vaccination against pneumococci after the operation, but patients who had a definite diagnosis for splenic injury preoperatively, and were scheduled for partial splenectomy, received vaccination before the surgery.

### 3.4. Follow up

Oral penicillin was prescribed as a prophylactic antibiotic for all patients after hospital discharge. The patients were evaluated three months after the surgery using a liver-spleen TC99 scan, complete blood count, and blood smear. The prophylactic antibiotic was discontinued if the spleen was reported as active. All study patients were followed for at least 6 months after the surgery.

### 3.5. Analysis

The data were registered and analyzed using SPSS V.14. The results are discussed using frequencies and mean values. Relative tables are also presented.

## 4. Results

Twenty patients with multiple traumas including injury to the spleen underwent partial splenectomy during 2006 to 2009 at our study centers. The average age of the patients was 20.15 years and 80% (n = 16) were male (Table 2). 

**Table 2.** Demographic Data and the Amount of the Salvaged Spleen in Patients

Patient Number	Age	Sex	Salvaged Spleen Percentage
**1**	7	♀	30
**2**	19	♂	40
**3**	20	♂	70
**4**	18	♂	70
**5**	15	♂	30
**6**	18	♂	50
**7**	6	♂	30
**8**	25	♂	60
**9**	4	♂	60
**10**	32	♂	60
**11**	17	♂	70
**12**	6	♀	60
**13**	35	♂	30
**14**	8	♀	30
**15**	20	♂	75
**16**	40	♂	30
**17**	5	♀	30
**18**	54	♂	30
**19**	18	♂	80
**20**	36	♂	30

Mechanisms of trauma included car accidents (n = 9), motorcycle accidents (n = 5), falls (n = 4), and stab wounds (n = 2). Seven patients had additional intraperitoneal organ injuries, while extraperitoneal injuries such as haemothorax, mandibular fracture, and tibia and fibula fractures were also present in some patients (Table 3). 

**Table 3.** Associated Intra- and Extraperitoneal Injuries in 10 Patients.

Injury	Patient Number	Treatment
**Diaphragm laceration**	1	Simultaneous surgical repair with partial splenectomy
**Liver injury, stage I and II**	2	Simultaneous surgical repair with partial splenectomy
**Peritoneal bleeding in the left zone II**	3	Supportive treatment
**Haemothorax**	4	Thoracostomy tube insertion
**Fracture of the mandible**	1	Surgical treatment after partial splenectomy
**Fracture of the tibia and fibula**	2	Surgical treatment after partial splenectomy
**Pancreatic hematoma**	2	Supportive treatment
**Small intestine and mesenteric hematoma**	1	Supportive treatment

The surgery (partial splenectomy and other necessary operations) lasted less than two hours for 15 (75%) patients and between two to three hours for 5 (25%) patients. The hospitalization period was between three to five days for 11 (55%) patients, seven to nine days for seven (35%), and 10 to 11 days for two (10%) patients. The shortest hospitalization period was three days and the longest was 11.

No early or late complications were reported following partial splenectomy during the hospital stay or after discharge. A liver-spleen Tc99 scan was performed for all patients three months after the surgery. Radioactive uptake in the spleen was reported for all patients. In cases for which more than 50% of the spleen had been preserved, an irregular border was observed in the spleen scan. The Tc99 scan was reported as faint and minimal in patients for whom 30% of the spleen was salvaged. The blood smear of 15 (75%) patients was normal three months after the operation, while 5 (25%) patients showed changes, which are described in Table 4. In addition, white blood cell and platelet counts obtained before the surgery and three months later are presented in Table 4. 

**Table 4.** White Blood Cell and Platelet Count and Blood Smear Results Before Partial Splenectomy and 3 Months After

Patient Number	Platelet		White Blood Count		Blood Smear	
	Pre-op	Post-op	Pre-op	Post-op	Pre-op	Post-op
**1**	419000	371000	16100	9100	Normal	Normal
**2**	197000	282000	9200	9700	Normal	Normal
**3**	148000	244000	21000	11700	Normal	Normal
**4**	282000	419000	13700	5100	Normal	Normal
**5**	172000	289000	12600	10900	Normal	Anisocytosis, Poikilocytosis, Hypochromia, Reticulocytes: 0.3
**6**	130000	237000	11700	9000	Normal	Normal
**7**	252000	265000	25300	6400	Normal	Normal
**8**	110000	342000	14100	14500	Normal	Hypersegmented neutrophils
**9**	210000	250000	18000	12500	Normal	Giant platelet Hypersegmented neutrophil
**10**	150000	160000	9400	9600	Normal	Normal
**11**	350000	613000	18000	7700	Normal	Normal
**12**	127000	382000	5900	7400	Normal	Normal
**13**	150000	70000	18800	12700	Normal	Giant platelets, Hypersegmented neutrophils, Anisocytosis
**14**	150000	220000	11700	10000	Normal	Normal
**15**	228000	211000	23500	7530	Normal	Normal
**16**	273000	182000	22000	5500	Normal	Howell-Jolly bodies
**17**	250000	390000	9500	11900	Normal	Normal
**18**	180000	280000	13900	9400	Normal	Normal
**19**	233000	208000	15000	7500	Normal	Normal
**20**	173000	274000	21900	7800	Normal	Normal

Sixteen patients (80%) were followed for 2 years after the surgery and did not receive any vaccination or antibiotic therapy during this period. Partial splenectomy was performed in 2009 for 4 patients; they were followed for 6 months after the operation and no complication was reported.

## 5. Discussion

The spleen is the most commonly injured solid organ in multiple traumas (1, 2). Total splenectomy used to be considered as the standard approach for an injured spleen but the high risk of overwhelming post-splenectomy infections in splenectomized patients has changed the general attitude towards splenectomy. A study on 320 patients with splenic traumas in Iran also showed that there is an increasing trend towards splenic preservation, particularly in younger, stable patients (15). In addition to vaccination against meningococci, Haemophilus influenzae, and pneumococci, splenectomized patients should receive oral penicillin for several years after the surgery (16). Due of the important role of the spleen in the immune system and fatal complications of total splenectomy, since the 1960s, partial splenectomy has received more attention and has become the preferred treatment for the injured spleen (17).

Partial splenectomy can be performed by laparoscopic techniques (18, 19). However, the laparoscopic approach is more frequently used for non-traumatic indications and elective operations (19). In trauma patients, laparoscopy is uncommon due to the unstable hemodynamic conditions of the patient. Laparoscopic splenectomy for a ruptured spleen has been reported only in for few cases, in which a hand-assisted technique was used (20). Therefore, in our study all the patients underwent laparotomy.

Spleen tissue contains an abundance of vessels so bleeding is a major problem in partial splenectomy. To perform partial splenectomy, vascular isolation techniques are usually applied. In addition, arteries supplying the injured area are usually ligated before resection using various methods such as ligature, stapling, suturing, cauterization, and radiofrequency (18, 21-24). Although hemostasis techniques are applied, bleeding still remains a major problem for partial splenectomy, which encourages surgeons to perform total splenectomy for multiple trauma patients, especially in cases with associated intraperitoneal injuries or unstable hemodynamic situations. In our study, 50% of the patients had associated intra- and extraperitoneal injuries such as diaphragm laceration, grade I and II liver injury, and kidney injury in addition to the spleen injury. All mentioned associated injuries were treated at the same time as the partial splenectomy and no further complications were reported.

Successful partial splenectomy relies on hemodynamic stability of the patient, especially during anesthesia, and sufficient hemostasis for the spleen and other injured organs. It is recommended not to perform partial splenectomy when extensive injuries to organs such as the liver, pancreas, and prostate are present (2). These injured organs can release a large amount of plasminogen that causes hemostatic dysregulation and bleeding. We did not use vascular isolation techniques for partial splenectomy, because we believe that it could increase the risk of splenic vascular damage during the surgery, as well as prolonging the time of the operation. Our technique included the resection of damaged splenic tissue only. In cases of hilar artery ligation, the blood supply of the upper and lower splenic margins is provided by short gastro and left gastric epiploic arteries along with branches of the splenic artery proximal to the splenic hilum.

Several techniques have been introduced for hemostasis in the resected area of the spleen (16, 22, 24). In our study, after the resection of damaged tissue with wedge shaped incisions hemostasis was established by applying two rows of 2/0 chromic sutures. The first row was applied through the spleen by continuous mattress sutures and resulted in relative hemostasis at the base of the resected area. The second row of sutures enclosed the incised wedge edges and completed hemostasis by compressing the intraparenchymal vessels. During the surgery, the assistant surgeon manually compressed the upper and lower cut edges of the spleen, which reduced excessive tension of the sutures on spleen tissue and prevented subsequent ischemia or parenchymal tearing.

The Penrose drain was used for the following objectives: first, to drain seeping plasma or blood pooled at the resected area; second, to prevent collection of fluid under the diaphragm; and finally, for early diagnosis of bleeding from the repaired edge of the spleen. No complications related to the insertion of Penrose drains occurred in our study.

In 75% of the patients the operation took less than two hours. In the remaining patients the operation took two to three hours, which was in all cases related to the management of associated organ injuries. The operation time and hospital stay was similar to that of patients who underwent elective non-emergent partial splenectomy (18, 23). It seems that the surgeon’s expertise has a tremendous role in decreasing the time of the surgery.

A liver-spleen Tc99 scan is the standard procedure for the evaluation of spleen function (17, 19). The scan results reported radioactive uptake in all 20 patients from our study, which indicates the functionality and viability of the salvaged spleen. In addition, the complete blood counts and blood smears obtained were normal for 15 patients, which confirms the scan results. Total splenectomy could possibly result in increased platelet counts and leukocytosis from several weeks to months after the surgery (2). Various factors such as infection, inflammation, and other stresses may also affect the white blood cell and platelet count. Although minor changes were observed in the white blood cell and platelet count of 25% of patients, the values were still in the normal range and it seems that the changes may not have had a direct relationship with partial splenectomy. These patients demonstrated viable spleen tissue on the Tc99 scan. The salvaged spleen tissue was 30% for three and 60% for two of the five mentioned patients. The first three patients had major spleen tissue resection. This might have affected the organ functionality for up to three months after the surgery. However, six months after the surgery the spleen function had returned to normal in these patients which could have been related to spleen regeneration. It seems that a specific amount of spleen tissue is required for an individual to maintain normal physiologic function of the spleen. This amount is reported to be 10 to 15% by some studies, but the majority of recent studies report the minimum amount of spleen tissue required for its functionality is 25 to 30% (17, 19). Furthermore, the decreased function of the spleen in the first three patients may have been due to the imbalance between the amount of salvaged spleen tissue and the patients’ weight. On the other hand, we had two patients with decreased splenic function in spite of an approximate 60% preservation of spleen tissue; one of them was a four year old child and the other was a 32 year-old-man. The decreased functionality in these patients may have been due to a preexisting splenic malfunction prior to the trauma, which was exaggerated by the resection of 40% of the spleen tissue. The complete blood count and blood smear became normal after six months in the four-year-old child. The 32-year-old man, however, still demonstrated an abnormality in complete blood count, but was healthy without any vaccination or antibiotic therapy after 2 years of follow up visits. A study in Japan comparing the immunologic alteration and long-term prognosis after splenic injury with preservation treatment (embolization, splenorrhaphy, partial splencetomy), showed that there was no significant difference in serum levels of IgM or specific IgG antibodies against 14 types of *Streptococcus pneumoniae* capsular polysaccharide, C3, C4, high-sensitivity C-reactive protein, and B - cell subset (surface marker immunoglobulins: IgA, IgG, and IgM) (15).

Several strong points can be derived from the results of our study. This surgical technique is simple, fast, without vascular isolation, usable for all age groups, does not need praphylaxis post operatively and there is no need for expensive hemostatic material or procedures. This method can be used for injury to upper, lower and central portion of the spleen without hilar in injury. Splenic function was preserved after the surgery.

Experience of the surgeon is important for using of this technique. Also we didn’t use this method for the excluded patients that were mentioned previously in the metholdology. These patients had risk factors for which they were considered to have poor conditions. Reoperation of these patients following failed partial spelenectomy increases the rate of mortality.

In conclusion, partial splenectomy can be performed for a hemodynamically stable multiple trauma patient whose splenic injury is not higher than stage IV. In addition, hemostasis can be safely achieved using two rows of 2/0 chromic sutures on the resected spleen edges. A Penrose drain can also be used as a safe method for drainage. It is recommended to salvage at least 30% of the splenic tissue in partial splenectomy to retain normal function of the spleen.
